# The Impact of Bariatric Surgery on Menstrual Abnormalities—a Cross-Sectional Study

**DOI:** 10.1007/s11695-020-04840-6

**Published:** 2020-07-13

**Authors:** Anna Różańska-Walędziak, Paweł Bartnik, Joanna Kacperczyk-Bartnik, Krzysztof Czajkowski, Maciej Walędziak

**Affiliations:** 1 2nd Department of Obstetrics and Gynecology, Medical University of Warsaw, Karowa 2 St., 00-315 Warsaw, Poland; 2 Department of General, Oncological, Metabolic and Thoracic Surgery, Military Institute of Medicine, Szaserów 128 St., 04-141 Warsaw, Poland

**Keywords:** Bariatric surgery, Menstrual cycle, Menstrual disturbances, Obesity

## Abstract

**Introduction:**

Obesity is associated with hyperestrogenism along with other hormonal abnormalities affecting the menstrual cycle. The most effective and decisive method of obesity treatment is bariatric surgery. The aim of this study was to analyze the impact of bariatric surgery on menstrual cycle, the incidence of menstrual abnormalities, hyperandrogenism manifestation, and contraception use.

**Materials and Methods:**

It was a cross-sectional study of 515 pre-menopausal women who had undergone bariatric surgery between 1999 and 2017 in a bariatric center. Data was collected via anonymous questionnaire, and the questions covered a 1-year period before the surgery and the last year before questionnaire completion.

**Results:**

Before the surgery, 38.6% of the patients reported irregular menstruations in comparison with 25.0% after bariatric surgery (RR = 0.65; 95%CI 0.53–0.79). The mean number of menstruations per year did not differ before and after surgery (10.2 ± 3.9 vs 10.4 ± 3.3; *p* < .45). There were no statistically significant differences in terms of prolonged menstruations, acne, and hirsutism prevalence. A total of 14.4% of patients before surgery reported estrogen-based contraception use in comparison with 15.0% after the surgery (*p* < .95). There were no significant differences in the frequency of OC use (11.0% before surgery vs 13.6% 12 months after the surgery vs 11.5% at the moment of survey administration; *p* < 0.46).

**Conclusion:**

Bariatric surgery improves the regularity of the menstrual cycle in obese women in reproductive age. The lack of any changes in the combined hormonal contraception (CHC) use, especially OC, before and after bariatric surgery may be a result of a possibly low level of contraception counseling.

## Introduction

The prevalence of obesity among women in Europe has been steadily increasing during the last 2 decades [1]. Obesity is associated with an increased risk of several chronic diseases and increases general mortality [2]. What is more, it has a negative impact on the overall life quality [3]. In addition to having greater risks of cardiovascular disease and diabetes mellitus, obese women are more likely to experience sequelae of reproductive health issues including polycystic ovarian syndrome (PCOS) and infertility [4–6].

Obesity is associated with hyperestrogenism along with other hormonal abnormalities affecting the menstrual cycle [7]. Obese women suffer more frequently from menstrual cycle abnormalities; their menstruation is longer and heavier [7]. As there is a strict association between obesity and PCOS incidence, obese women have a higher risk of presenting hyperandrogenic features, such as acne or hirsutism [5]. Obesity should be considered while choosing the appropriate contraception method, as it affects both the pharmacokinetics and the risk of thromboembolic events [8, 9].

The most effective and decisive method of obesity treatment is bariatric surgery (BS) [10]. It includes various surgical procedures, e.g., sleeve gastrectomy, Roux-en-Y gastric bypass, or adjustable gastric band [2]. It is, however, associated with long-lasting side effects for the patients. Most of them require lifelong microelement supplementation, and most techniques are irreversible [2]. BS affects the efficiency of contraception, especially oral contraception (OC) [11].

There are a few studies on the effects the bariatric surgery on the reproductive endocrinological status of the operated women [4–6, 12–19]. Most of them analyze the effects of BS on fertility or focus on laboratory hormonal status [12, 13, 18]. Few, however, focus on clinical effects, and their results are often colluding.

The aim of this study was to analyze the impact of bariatric surgery on menstrual cycle, the incidence of menstrual abnormalities, hyperandrogenism manifestation, and contraception use.

## Material and Methods

It was a cross-sectional study of 515 patients who had undergone bariatric surgery between 1999 and 2017 in a bariatric center and were pre-menopausal at the moment of survey administration. The exclusion criteria were postmenopausal status at the moment of survey administration, history of more than one bariatric procedure, adjustable gastric band (AGB), and lack of data essential for the study, e.g., type of surgery. It was a part of a bigger project analyzing the effects of bariatric surgery on the course of pregnancy and female sexual function [20].

Patients were recruited on the basis of medical records. All women underwent the sleeve gastrectomy (SG) or Roux-en-Y gastric bypass (RYGB). All participants met the Interdisciplinary European Guidelines on Metabolic and Bariatric Surgery criteria for bariatric surgery [21]. Each patient was contacted via telephone and invited to complete a questionnaire. The questions covered a period of 1 year before the surgery and the last year before questionnaire completion. Each patient was contacted at least 3 times in the 48-h period before considered unapproachable. Patients completed the questionnaires anonymously via an online questionnaire, paper version of the questionnaire, or through a telephone conversation with a medical doctor. The method of questionnaire completion depended on patient’s preferences. The initial number of patients contacted was 1001, 623 out of whom completed the questionnaire. The response ratio was 62.3%.

The questions about the menstrual cycle and other reproductive gynecological issues considered the following:

- The absolute number of menstruations during 1 year
- The span of the menstrual cycle, where cycles occurring between 25 and 35 days were considered normal [22]
- The average time of menstruation, where menstruations longer than 7 days were considered prolonged [22]
- Acne presence
- Hirsutism presence
- Use of contraception methods with the distinction of the estrogen-based methods

Statistical analysis was performed using Statistica 13 (StatSoft. Inc.). Student’s *t* tests were used for quantitative data comparison between two groups. Two-sided Fisher’s exact test and chi-square test were used for categorical and binary data comparison as required. *p* value < .05 was considered significant.

## Results

The group characteristics are presented in Table 1. The mean age at the point of questionnaire completion was 37.4 ± 7.7 years. The median time from surgery to survey was 37.4 months. The majority, 70.3% of patients, underwent sleeve gastrectomy, while 29.7% underwent Roux-en-Y gastric bypass. The mean excess weight loss (EWL) was 74.0 ± 30.4%. A total of 82.0% of the patients achieved a 50% EWL.

**Table 1:** Group characteristics

Feature	Value
Age (at interview)	37.4 ± 7.7
Time from surgery (median; 1st–3rd quartile) (months)	37.4 (25.2–53.5)
Pre-surgery BMI (mean ± SD) (kg/m^2^)	42.2 ± 7.5
Pre-surgery weight (mean ± SD) (kg)	121.0 ± 19.9
Current BMI (mean ± SD) (kg/m^2^)	29.8 ± 6.3
Current weight (mean ± SD) (kg)	85.2 ± 18.0
BMI loss (mean ± SD) (kg/m^2^)	12.4 ± 6.3
Weight loss (mean ± SD) (kg)	35.3 ± 17.9
Total weight loss (mean ± SD; %)	29.4 ± 10.7%
Excess weight loss (mean ± SD; %)	74.0 ± 30.4%
Patients who achieved 50% excess weight loss (%)	386 (82.0)
Residency (%)	
- Village	95 (20.2)
- Town < 20,000 inh.	36 (7.6)
- Town 20,000–100,000 inh.	102 (21.7)
- City (over 100,000 inh.)	238 (50.53)
Education (%)	
- Primary	7 (1.5)
- Secondary	204 (43.3)
- Higher	260 (55.2)
Currently employed (%)	376 (79.7)
Type of surgery (%)	
- Sleeve gastrectomy	332 (70.3)
- Roux-en-Y gastric bypass	140 (29.7)

Table 2 presents the comparison between the menstrual abnormalities, hirsutism, acne, and estrogen-based contraception use before and after the surgery. Before the surgery, 38.6% of the patients reported irregular menstruations in comparison with 25.0% after the bariatric surgery (*p* < .001) (RR = 0.65; 95%CI 0.53–0.79). The mean number of menstruations per year did not differ before and after surgery (10.2 ± 3.9 vs 10.4 ± 3.3; *p* < .36). There were no statistically significant differences in terms of prolonged menstruations, acne, and hirsutism prevalence. A total of 14.4% of patients before surgery reported estrogen-based contraception use in comparison with 15% both 12 months after the surgery and at the moment of survey administration (*p* < .95). There were no significant differences in the frequency of OC use (11.0% before surgery vs 13.6% 12 months after the surgery vs 11.5% at the moment of survey administration; *p* < 0.46). The use of other types of contraception is presented in Table 2.

**Table 2:** Results

**Feature**	**Pre-operative**	**Postoperative**	***p***	
Irregular menstruation prevalence (%)*	181 (38.6)	117 (25.0)	< .001	
Prolonged menstruation (%)**	58 (12.4)	40 (8.5)	< .06	
Number of menstruations per year (± SD)	10.2 ± 3.9	10.4 ± 3.3	< .36	
Acne prevalence (%)	142 (30.1)	121 (25.7)	< .13	
Hirsutism prevalence (%)	162 (34.5)	159 (33.8)	< .84	
	**Before surgery**	**12 months after surgery**	**At the moment of survey administration**	***p***
Contraception use (any; %)	286 (60.6)	284 (60.2)	268 (56.8)	< .43
Barrier methods (%)	178 (37.7)	192 (40.7)	160 (33.9)	< .10
IUD (copper; %)	21 (4.4)	18 (3.8)	23 (4.9)	< .73
IUD (hormonal; %)	25 (5.3)	20 (4.2)	30 (6.4)	< .35
Estrogen-based contraception use (any; %)	68 (14.4)	71 (15.0)	71 (15.0)	< .95
- Oral contraception (%)	52 (11.0)	64 (13.6)	55 (11.5)	< .46
- Vaginal rings (%)	9 (1.9)	5 (1.1)	13 (2.7)	< .16
- Patches (%)	9 (1.9)	5 (1.1)	4 (0.9)	< .31

*Irregular menstruations were defined as menstruations occurring outside the interval of 25 to 35 days

**Prolonged menstruations were defined as menstruations occurring longer than 7 days

## Discussion

The first observation made in the study was the significant increase in the number of patients with regular menstruations after BS without the impact on the absolute number of menstruation cycles. Most of the similar studies confirm this observation [5, 6, 13, 16, 17, 19]. Many of the studies analyze the menstruation improvement in the selected population of obese patients with PCOS [5, 12, 13]. It is a very positive observation that the observed improvement of 35% concerned the whole analyzed population, without the distinction of PCOS patients. In the PCOS patients, the lowering of the BMI has a strong and well-documented association with menstrual pattern improvement [5]. In obese patients without PCOS, such a relationship is much weaker. Therefore, the high chances of improvement of the regularity of the menstruations, with or without the diagnosis of PCOS, seem to have its place in the counseling before the BS.

We did not observe any significant differences before and after BS in terms of prolonged (> 7 days) menstrual bleeding. The only study obtained by the authors shares similar observations [6]. The relatively high proportion (12.4%) in comparison with the general population before the surgery seems to possibly be the effect of hyperestrogenism caused by obesity. In our study, unfortunately, the heaviness of menstrual bleeding was not analyzed in order not to create perception bias. It is possible that the reduction of hyperestrogenism caused by excessive weight loss could lead to not only more regular but also less heavy menstruations.

We did not observe any improvement in the clinical manifestations of hyperandrogenism prevalence—acne and hirsutism—before and after BS. Studies analyzing the effects of BS on incidence of these symptoms in the PCOS population showed a significant beneficial effect of BS [13, 23]. Christ and Falcone compared a group of PCOS and non-PCOS patients after BS and found a higher decline in the androgen levels in the PCOS group. Patients from the PCOS group had higher androgen levels at the baseline, and after BS, their DHEAS and free testosterone levels did not differ from the controls. The decrease in androgen levels after BS is also associated with an increase of sex hormone binding globulin (SHBG). The more significant reduction in androgen level may explain the more beneficial effects on reduction of acne and hirsutism in the PCOS group. However, the study by Legro et al., which analyzed the population without distinction on those with or without PCOS, observed a similar lack of beneficial effects of BS on the clinical manifestation of hyperandrogenism as in our study [17]. As the average time of questionnaire completion is approximately 3 years after the BS, if such an improvement occurred, it would have been observed. Therefore, it is likely that BS has a most beneficial effect in terms of hyperandrogenism improvement only in patients with PCOS.

The next interesting aspect of this study is the use of different birth control methods in the analyzed group. It has been established that the use of estrogen-based type of contraception in obese patients has a steady negative impact on the thromboembolic event risk [9]. The UK MEC advises against combined hormonal contraception (CHC) use in women with a body mass index (BMI) ≥ 35 kg/m^2^ [24]. In addition to this, the use of most popular form of CHC, oral contraception, is associated with high risk of suboptimal efficiency, mostly due to malabsorption of medicines administered orally [11]. The observation made during the study as the approximately 15% of patients both before and after the surgery used CHC seems to be alarming. This proportion, despite being relatively low in comparison with other studies, does not change after BS. Similar observations were made in the USA [18]. The studies by Ginstman et al. and Luyssen et al. provided different observations, as in their studies, patients after BS used CHC less often [6, 14]. The fact that such observation was not made leads to a suspicion that BMI was taken into account to a minimal level during contraception counseling in this group of patients. What is more, the majority of analyzed patients used hormonal OC and there were no significant differences in its use before and after surgery. The study of Damhof et al. which classified use of OC after BS as “unsafe” showed significant, nearly 2-fold, reduction of OC after BS [25]. According to the consensus recommendations by Shawe et al., patients with the history of bariatric surgery require appropriate safe and effective contraception, especially during the rapid weight loss period [26]. According to the recommendations, absorption of orally administered contraceptives can be compromised and other methods of birth control should be advised [26]. Although the guidelines discourage use of combined oral contraception (COC) in patients after BS, there is a lack of level 1 evidence on the decreased absorption of oral estrogens after BS. The actual reliability of COC after currently used BS procedure has not been sufficiently analyzed, and guidelines are based on pharmacokinetic studies that analyzed older types of BS like jejunoileal bypass. There are some new studies suggesting normal pharmacokinetics of etonogestrel after BS, and the subject of estrogen and progestagen bioavailability after BS should be subject to further studies [27]. The problem of optimum postoperative contraceptive methods should be discussed with the patients as pre-operative counseling plays a major role in the postoperative gynecologic care. Mengesha et al. have observed that even one perioperative contraception counseling has a positive effect on adequate contraception use [28].

## Limitations of the Study

The main bias source in our study comes from its cross-sectional nature. Patients reported characteristics of their menstrual cycle in a retrospective manner, which additionally may be the source of bias. In addition to this, the study lacks the question about the perceived blood loss. It was, however, not added by the authors due to possible heavy perception bias if patients reported it without verification.

## Conclusions

Bariatric surgery improves the regularity of the menstrual cycle in obese women in reproductive age. In the general obese population, without the distinction of the patients with the PCOS, it does not seem to have a positive impact on the clinical hyperandrogenism manifestation. The lack of any changes in the CHC use, especially OC, before and after bariatric surgery may be a result of a possibly low level of contraception counseling.
